# Humanized hiPSC Platforms for I/R Injury: Advancing Toward Precision Cardioprotection

**DOI:** 10.1155/cdr/2729114

**Published:** 2026-09-15

**Authors:** Mengyan Mi, Tzu-Yu Chen, Honghua Ye

**Affiliations:** ^1^ Ningbo University, Ningbo, Zhejiang, China, nbu.edu.cn; ^2^ Department of Cardiology, Ningbo Medical Centre Lihuili Hospital, Ningbo, Zhejiang, China; ^3^ Science and Technology R&D Department, Ningbo No. 2 Hospital, Ningbo, Zhejiang, China, nbws.gov.cn

**Keywords:** cardiac organoids, ferroptosis, heart-on-a-chip, human induced pluripotent stem cells, mitochondrial dynamics, myocardial ischemia–reperfusion injury, precision cardiology, single-cell multiomics, spatial transcriptomics

## Abstract

Myocardial ischemia–reperfusion (I/R) injury remains a major contributor to infarct expansion, adverse remodeling, and heart failure despite timely coronary revascularization. The repeated failure of cardioprotective interventions that were effective in animal models has exposed a persistent translational gap. Human induced pluripotent stem cell–derived cardiomyocytes (hiPSC‐CMs) and engineered cardiac platforms preserve donor‐specific human genetic backgrounds, provide scalable cell sources, and offer experimentally tractable systems for studying selected mechanisms of I/R injury. Recent advances in electrical, mechanical, metabolic, and endocrine maturation, together with engineered heart tissues (EHTs), cardiac organoids, and perfused heart‐on‐a‐chip platforms, have improved the ability to model cardiomyocyte stress, calcium overload, mitochondrial dysfunction, oxidative injury, and multicellular crosstalk. Single‐cell and spatial omics, CRISPR‐based perturbation, and artificial intelligence (AI)–assisted high‐content phenotyping further enable state‐resolved and mechanistically testable analyses of vulnerable cardiac cell populations. In this narrative review, we synthesize recent progress in hiPSC‐based myocardial I/R modeling, critically evaluate the strengths and limitations of current platforms, and discuss their use in mechanism‐guided drug screening, cardiotoxicity assessment, and patient‐specific preclinical modeling. We emphasize that these systems are not yet substitutes for clinical validation or whole‐organ physiology. Their current value lies in providing controllable, human‐relevant preclinical models that can prioritize mechanisms, identify candidate interventions, and support better‐designed translational cardioprotection studies.

## 1. Introduction

Timely reperfusion therapy after acute myocardial infarction (AMI) remains the cornerstone of myocardial salvage. However, the resulting myocardial ischemia–reperfusion (I/R) injury substantially attenuates these benefits by promoting infarct expansion, adverse ventricular remodeling, and progression to heart failure. Despite decades of intensive investigation, clinically effective cardioprotective strategies for I/R injury remain limited, leaving a major unresolved challenge in cardiovascular therapeutics.

This persistent translational impasse highlights a central gap in I/R injury research. A major contributing factor is the continued reliance of preclinical studies on animal models, whose predictive value is compromised by substantial interspecies differences in cardiac physiology, metabolism, electrophysiology, immune responses, and gene regulatory programs [1, 2]. Conventional in vitro systems, including immortalized cardiomyocyte cell lines and primary human cardiomyocytes, are likewise constrained by nonphysiological features, limited availability, poor scalability, and limited capacity for repeated mechanistic perturbation. These limitations underscore the need for human‐relevant experimental platforms that can model selected components of human I/R injury under controlled and reproducible conditions.

Human induced pluripotent stem cell (hiPSC) technology provides one approach to addressing these bottlenecks [3]. hiPSC‐CMs retain the donor′s human genomic background and can, therefore, reduce, although not eliminate, the interpretive limitations imposed by interspecies divergence. Their self‐renewal capacity and amenability to directed differentiation help address the scarcity of primary human cardiomyocytes and provide a scalable foundation for disease modeling [4]. Directed differentiation based on temporally staged biphasic modulation of Wnt/*β*‐catenin signaling can generate cardiomyocytes at high purity [5, 6], whereas stirred suspension and bioreactor workflows have improved large‐scale hiPSC‐CM production for screening and tissue engineering [7, 8].

However, early two‐dimensional hiPSC‐CM monolayer models exhibit a pronounced fetal‐like and metabolically immature phenotype, characterized by a predominant reliance on glycolysis [9, 10]. This immaturity limits their ability to recapitulate adult cardiomyocyte responses to ischemic stress [11, 12]. In response, the field has rapidly moved toward more sophisticated platforms, including engineered three‐dimensional tissues [13], multicellular cardiac organoids [14], and microfluidic heart‐on‐a‐chip systems [15], which improve pathophysiological relevance by recreating cell–cell interactions and spatial organization [16]. A particularly important current trend is the systems‐level integration of organoids and other microphysiological models with microfluidics and precisely controlled electrical and/or mechanical stimulation [17, 18]. For example, multicellular heart‐on‐a‐chip microphysiological systems incorporating endothelial cells, fibroblasts, and hiPSC‐CMs can introduce controlled microenvironmental cues and cell–cell interactions relevant to myocardial I/R modeling [18]. Collectively, these multidimensional maturation strategies and bioengineering advances converge on a common goal: to overcome the limitations imposed by cellular immaturity and missing microenvironmental cues and to establish more controlled and human‐relevant experimental platforms for I/R injury research.

In parallel, single‐cell multiomics, spatial transcriptomics (ST), and CRISPR–Cas9‐based genome editing are reshaping the understanding of I/R injury as a heterogeneous and state‐dependent process. These approaches enable higher‐resolution characterization of injury heterogeneity, spatial patterning, and causal regulatory mechanisms in human cells. However, they remain dependent on rigorous platform validation, standardized injury protocols, and careful interpretation of evidence boundaries.

In this review, we describe how hiPSC‐based I/R injury modeling has evolved from relatively simple hypoxia/reoxygenation (H/R) paradigms to more complex systems incorporating multicellular interactions, vascularization, and biomechanical stimulation. We examine how successive platform innovations address persistent challenges such as cardiomyocyte immaturity, insufficient tissue complexity, and limited functional readouts. We also evaluate limitations related to reproducibility, scalability, cost, standardization, and clinical translation; synthesize mechanistic insights generated by these models, including regulated cell death pathways such as ferroptosis; and discuss emerging applications in drug discovery and patient‐specific preclinical modeling. Finally, we outline future directions for next‐generation humanized cardioprotection platforms arising from the convergence of stem cell engineering, biofabrication, multiomics, and computational analytics.

### 1.1. Clinical Burden of Myocardial I/R Injury

**Figure 1:**
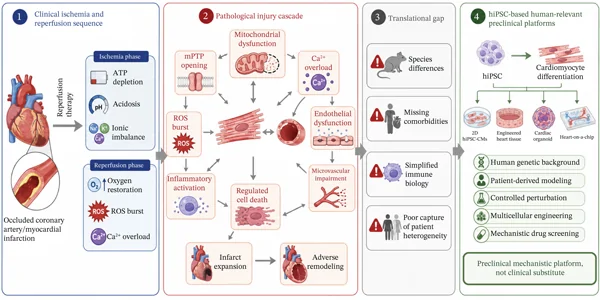
Pathological sequence of myocardial I/R injury and rationale for hiPSC‐based modeling. The figure separates the ischemic and reperfusion phases and annotates ATP depletion, acidosis, ionic imbalance, Ca^2+^ overload, ROS burst, mPTP opening, endothelial dysfunction, microvascular impairment, inflammatory activation, regulated cell death, infarct expansion, and adverse remodeling. It also highlights why conventional animal and simplified in vitro models incompletely capture human comorbidities, immune biology, and patient heterogeneity, thereby motivating human‐relevant hiPSC‐based platforms.

AMI remains a leading cause of death and disability worldwide [19]. Although early percutaneous coronary intervention (PCI) has markedly improved coronary reperfusion and reduced short‐term mortality, reperfusion itself can trigger myocardial I/R injury, which may account for up to approximately 50% of the final infarct size [20]. Preclinical studies have identified several core mechanisms of myocardial I/R injury, including oxidative stress, calcium overload, opening of the mitochondrial permeability transition pore (mPTP), inflammatory activation, and mitochondrial fission–related injury pathways [21–23]. However, pharmacological or procedural interventions targeting these mechanisms have repeatedly failed to show consistent clinical benefit. Representative examples include the mPTP‐targeting agent Cyclosporine A, ischemic postconditioning, and adenosine‐based interventions [24–26]. This series of negative or inconsistent clinical results has exposed a long‐standing translational gap between mechanistic discovery and clinically effective cardioprotection. Several factors have contributed to this problem (Figure 1).

### 1.2. Persistent Translational Failure of Cardioprotection Despite Timely Reperfusion

#### 1.2.1. Interspecies Divergence Between Animal Models and Human Myocardium

Rodent hearts differ substantially from human myocardium in heart rate, ion channel expression, substrate preference, mitochondrial density, and transcriptional regulatory architecture [27]. Murine myocardium relies more heavily on glucose utilization, whereas the adult human heart derives most of its ATP from fatty acid oxidation [28]. In addition, humans and mice show marked divergence in inflammatory gene regulation and innate immune signaling pathways [29]. As a result, mitochondrial, metabolic, and inflammation‐related mechanisms validated in mice do not necessarily translate to the human setting.

#### 1.2.2. Lack of Clinically Relevant Comorbidity Context in Animal Models

Most experimental studies are performed in young and otherwise healthy animals, whereas patients with AMI are often older and commonly present with diabetes, dyslipidemia, hypertension, and other comorbidities. These conditions can attenuate endogenous cardioprotective signaling and alter mPTP sensitivity [30]. Consequently, interventions that are effective in healthy animals may fail in real‐world patient populations.

#### 1.2.3. Oversimplified Modeling of Immune Responses

Inflammation is a major determinant of reperfusion injury and subsequent remodeling. However, rodents and humans differ substantially in inflammatory gene regulation, cytokine responses, immune cell composition, and temporal resolution of inflammation, which can limit the predictive value of animal models for human reperfusion‐associated immune injury [29]. Moreover, many animal models cannot fully recapitulate the complex interactions among cardiomyocytes, endothelial cells, fibroblasts, and immune cells, thereby limiting their predictive value.

#### 1.2.4. Inability to Capture Patient Heterogeneity

AMI is highly heterogeneous across individuals and is influenced by genetic background, sex, ancestry, and medication history [21]. Genetic polymorphisms affecting mitochondrial function, calcium homeostasis, and inflammatory signaling can markedly alter susceptibility to I/R injury and responses to cardioprotective therapies. Conventional animal models are poorly equipped to reproduce such patient‐specific variability.

### 1.3. Literature Search Strategy and Review Scope

This article is a narrative review rather than a systematic review or meta‐analysis. Relevant literature was identified through searches of PubMed, Web of Science, Scopus, and Google Scholar, with emphasis on studies published from January 2015 to July 9, 2026, and particular attention to work published from 2020 onward. Search terms included combinations of “human induced pluripotent stem cells,” “hiPSC‐CM,” “ischemia–reperfusion,” “hypoxia/reoxygenation,” “engineered heart tissue,” “cardiac organoid,” “heart‐on‐a‐chip,” “cardiomyocyte maturation,” “mitochondrial dysfunction,” “ferroptosis,” “mitochondria‐associated ER membranes,” “cGAS–STING,” “single‐cell RNA sequencing,” “spatial transcriptomics,” “CRISPR,” “high‐content imaging,” and “artificial intelligence.”

Studies were prioritized based on topical relevance, mechanistic importance, methodological novelty, and translational relevance to human myocardial I/R or H/R injury modeling. Priority was given to studies directly involving hiPSC‐derived cardiomyocytes, engineered cardiac tissues, cardiac organoids, microphysiological systems, or other human‐relevant experimental platforms. Additional non‐hiPSC, animal, or transplantation‐oriented studies were included when they provided mechanistic or translational context directly relevant to myocardial I/R injury, engineered cardiac tissues, ferroptosis, integrated functional assessment, or platform development. Because the review is narrative in scope, no formal risk‐of‐bias scoring, PRISMA‐based screening, or quantitative synthesis was performed. Evidence levels are, therefore, interpreted qualitatively, and clinical claims are deliberately framed as emerging possibilities unless supported by direct clinical validation.

## 2. Why hiPSC‐Based Platforms Are Needed for Myocardial I/R Modeling

Taken together, the limitations of traditional models largely arise from interspecies divergence, the absence of clinically relevant comorbidity contexts, oversimplified immune biology, and poor capture of interindividual heterogeneity. hiPSC‐based cardiac systems help address some of these barriers because they preserve human genetic background, can be generated from individuals with diverse genetic and clinical profiles, and can be engineered to include multiple cardiac lineages and selected immune components [17, 18]. Their value is, therefore, strongest when they are used as controllable human‐relevant systems for mechanistic testing, phenotype stratification, and candidate prioritization. At present, however, they should be considered complementary to animal models, human tissue studies, and clinical trials rather than replacements for whole‐organ or clinical validation.

### 2.1. Development of hiPSC‐Based Cardiac Disease Models

#### 2.1.1. From Human Embryonic Stem Cells (hESCs) to hiPSCs: Key Technological Milestones

The evolution of pluripotent stem cell–based cardiac modeling marks a major shift in cardiovascular research, moving from ethically constrained embryonic sources toward patient‐specific, genotype‐matched platforms. Early efforts in human cardiomyocyte differentiation relied predominantly on hESCs [31]. Their application was limited by ethical concerns, immune incompatibility, and restricted genetic diversity. The generation of iPSCs from somatic cells enabled pluripotent cell derivation without embryonic material while preserving donor‐specific genetic background, thereby laying a foundation for individualized disease modeling.

Initial embryoid body–based differentiation approaches were hampered by low efficiency and substantial batch‐to‐batch variability [32]. Monolayer differentiation paradigms based on biphasic, temporally controlled modulation of Wnt/*β*‐catenin signaling later improved efficiency and standardization [5, 33]. More recent progress has shifted from differentiation efficiency alone toward maturation, tissue architecture, and integrated functional assessment. These advances include engineered heart tissues (EHTs) using electrical and/or mechanical conditioning [34], endocrine maturation strategies [35], self‐organizing cardiac organoids, organ‐on‐a‐chip platforms, and biosensing‐compatible cardiac tissues [36–39]. Thus, hiPSC‐based cardiac models have matured from proof‐of‐concept systems into versatile preclinical platforms, although reproducibility, cost, and cross‐laboratory standardization remain major barriers.

#### 2.1.2. Directed Differentiation of hiPSC‐Derived Cardiomyocytes

Directed differentiation of hiPSC‐CMs has progressed from an early methodology‐driven stage focused primarily on differentiation efficiency to a standardized platform that integrates precise developmental signal control, scalable manufacturing, and translational robustness. At present, the canonical monolayer paradigm based on biphasic, temporally staged modulation of Wnt/*β*‐catenin signaling remains the core framework for cardiomyocyte induction and can reproducibly yield high‐purity cardiomyocyte populations.

Between 2022 and 2025, progress in this area shifted from conceptual refinement toward engineering optimization. Large‐scale differentiation workflows implemented in stirred suspension formats and bioreactor systems have substantially increased yield and improved cross‐line consistency while preserving the temporal precision of Wnt modulation, thereby enabling scalable cell production for high‐throughput drug screening and the construction of humanized I/R injury models [7]. Beyond lineage specification, control over cardiomyocyte purity and baseline metabolic state has emerged as a critical determinant of model interpretability. Notably, recent studies suggest that lactate‐based metabolic purification in two‐dimensional cultures can induce adaptive changes in metabolism and/or electrophysiology, resulting in ischemia‐like features. Such adaptations may confound baseline responses to ischemic stress and, therefore, require careful evaluation when studying energy metabolism–linked I/R injury mechanisms [40, 41]. Importantly, purification and enrichment strategies may influence baseline cell state and should, therefore, be reported when comparing 2D and 3D I/R models.

In addition, chamber‐ and region‐specific specification is of particular relevance to I/R injury research. Precise modulation of retinoic acid signaling during the transition from mesoderm to cardiac progenitors can selectively enrich atrial or atrial‐like hiPSC‐CMs [42]. Ventricular‐like cardiomyocytes more closely reflect the pathophysiological substrate of infarction and reperfusion injury, whereas atrial‐like cells provide a complementary model for studying ischemia‐associated atrial electrical remodeling and arrhythmogenesis. Taken together, contemporary differentiation strategies use temporal control of Wnt signaling as the core framework, with purification methods and chamber specification serving as tunable parameters that jointly define the basal state of hiPSC‐CMs. These features establish the cellular foundation for mechanistically interpretable, humanized I/R injury models.

#### 2.1.3. Maturation Strategies

hiPSC‐CMs have long been limited by a fetal‐like immature phenotype. Their predominantly glycolytic metabolic program differs fundamentally from the fatty acid oxidation–dominated metabolism of adult myocardium. This limitation directly compromises the translational value of humanized I/R injury models, because injury thresholds and phenotypic outputs in I/R injury depend heavily on robust mitochondrial oxidative metabolism, resilient calcium homeostasis, and frequency‐dependent electrophysiology. Accordingly, maturation strategies have evolved from single‐factor enhancement toward multidimensional integration designed to recapitulate key features of the postnatal cardiac developmental environment [43].

##### 2.1.3.1. Electrical Stimulation and Mechanical Loading

Chronic pacing can systematically improve structural maturation, electrophysiological properties, and calcium‐handling kinetics [44], thereby enhancing the reproducibility of reperfusion‐relevant phenotypes. Cyclic mechanical strain promotes sarcomere alignment and increases contractile force while reinforcing metabolic remodeling through mechanotransduction pathways [45, 46]. For I/R injury modeling, the integration of electrical and mechanical cues is especially important because it better captures the close interplay among reperfusion‐associated mechanical stress, calcium homeostasis, and the mitochondrial balance between energy supply and demand [45].

##### 2.1.3.2. Metabolic Reprogramming and Endocrine Maturation

Driving the metabolic transition from glycolysis toward fatty acid oxidation is central to metabolic maturation [46] and can simultaneously improve mitochondrial ultrastructure and function. In parallel, endocrine factors such as thyroid hormone, in combination with glucocorticoids, can promote electrophysiological maturation by modulating ion channel expression and rendering repolarization properties more adult‐like [35]. This maturation axis is particularly relevant to modeling reperfusion‐triggered arrhythmogenesis.

##### 2.1.3.3. Systems‐Integrated Platforms: Organoids and Perfused Chips

A major current trend is systems integration–driven maturation, in which organoids or microtissues are combined with microfluidic perfusion and programmable electrical and/or mechanical stimulation [38]. Such platforms not only provide dynamic nutrient delivery and biomechanical inputs that collectively promote tissue maturation but also, by virtue of their perfusability and vascular‐like architecture, serve as inherently suitable substrates for more physiologically relevant I/R injury modeling. For example, vascularized and perfusable human heart‐on‐a‐chip systems can model oxygen transport gradients, controlled perfusion, and ischemia‐related transport constraints, thereby improving analysis of spatiotemporal injury dynamics and drug delivery under I/R‐relevant conditions [47]. In addition, a 2025 *Nature Protocols* publication provided a detailed workflow for the standardized construction of these cardiac organ‐on‐a‐chip‐type systems [38].

**Figure 2:**
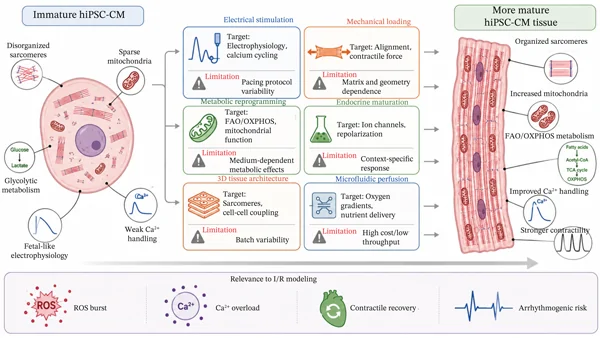
Systematic comparison of hiPSC‐CM maturation strategies for myocardial I/R modeling. The figure compares electrical stimulation, mechanical loading, metabolic reprogramming, endocrine maturation, 3D tissue architecture, and microfluidic perfusion. For each strategy, it indicates major maturation targets, advantages, limitations, and translational relevance to I/R injury modeling, including electrophysiology, calcium handling, mitochondrial oxidative metabolism, sarcomere organization, contractile force, and tissue‐level functional stability. FAO, fatty acid oxidation; OXPHOS, oxidative phosphorylation.

In summary, maturation strategies for I/R injury–oriented humanized platforms should be organized around the triad of mitochondrial energetics, calcium handling, and electrical stability. More informative systems increasingly combine metabolic reprogramming with electrical stimulation, mechanical loading, and microfluidic perfusion to improve electrophysiology and mitochondrial function. Nevertheless, even advanced maturation protocols rarely generate fully adult‐like cardiomyocytes; therefore, maturity status should be explicitly measured and reported when interpreting I/R injury thresholds and drug responses (Figure 2). For I/R modeling, maturation should not be treated as a generic improvement step. Instead, maturity status should be reported using mechanism‐relevant metrics, including mitochondrial respiration, fatty acid oxidation capacity, calcium transient kinetics, electrophysiological stability, sarcomere organization, and contractile force.

## 3. hiPSC‐Based Platforms for Modeling Myocardial I/R Injury

### 3.1. Experimental Platforms for Modeling Myocardial I/R Injury

#### 3.1.1. 2D H/R Systems: A Foundation for Mechanistic Dissection

Two‐dimensional H/R models represent the first generation of humanized I/R injury systems developed on hiPSC platforms. By reproducing hypoxia, substrate deprivation (typically glucose withdrawal), and reoxygenation in human cells, these models provide a tractable and tightly controlled framework for dissecting core mechanisms of reperfusion injury, most notably oxidative stress and calcium overload [48]. Chemical hypoxia approaches, including cobalt chloride–based methods, provide scalable hypoxia‐mimetic tools and have been used for early‐stage testing of candidate cardioprotective compounds in cardiomyocyte injury models [49, 50]. Because cobalt chloride creates a hypoxia‐mimetic state rather than true oxygen withdrawal and reoxygenation, findings from chemical hypoxia assays should be interpreted as scalable screening evidence and ideally validated in oxygen‐controlled hiPSC‐CM H/R systems.

Recent advances have moved 2D systems beyond simple viability‐based readouts toward high‐content, mechanism‐oriented platforms [51]. Through the integration of minute‐scale oxygen switching, multiparametric functional measurements, such as electrophysiology, calcium imaging, mitochondrial reactive oxygen species (ROS) readouts, and transcriptomic profiling, contemporary 2D H/R models can resolve temporal phases of functional recovery after reoxygenation and delineate mechanistic cascades linking metabolic reprogramming to mitochondrial dysfunction and cell death. Their high degree of standardization, relatively low cost, and compatibility with high‐throughput formats make them suitable for systematic pathway screening and initial prioritization of candidate cardioprotective compounds. Nevertheless, structural simplification remains an inherent limitation of 2D models. They lack physiologically relevant extracellular matrix architecture, multicellular paracrine networks, vascular components, immune cell recruitment, and biomechanical loading cues, all of which regulate I/R injury in vivo [52]. Accordingly, the principal value of 2D systems lies in providing a controllable reductionist platform for resolving subcellular injury pathways and generating hypotheses for testing in more complex three‐dimensional systems.

#### 3.1.2. 3D EHTs: Bridging Structure and Function

Three‐dimensional EHTs improve physiological relevance by embedding hiPSC‐CMs within hydrogel or scaffold‐free matrices and applying mechanical constraints that are absent in monolayer systems. Sustained mechanical loading promotes longitudinal alignment, sarcomere organization, electromechanical coupling, mitochondrial development, and calcium‐handling maturation compared with 2D culture systems [53]. Quantitative measurement of contractile force, for example, through post deflection, flexible electronics, or optical motion analysis, provides functional endpoints with mechanistic and translational relevance for I/R injury research [54]. Recent composite tissue sheet systems combining hiPSC‐CMs with human adipose‐derived stromal cells have also illustrated how multicellular tissue engineering can improve cardiac function in myocardial injury models, supporting the translational relevance of engineered human cardiac tissues while also highlighting the need to distinguish repair‐oriented transplantation models from in vitro I/R platforms [55].

In the context of I/R injury modeling, EHTs have progressed from descriptive phenotyping to more standardized platforms for functional assessment. Studies using EHT H/R or simulated I/R paradigms have shown contractile impairment and used contractile recovery after reoxygenation as a functional endpoint for pharmacological assessment, including *δ*‐opioid receptor agonists [56, 57]. Disease‐relevant metabolic contexts, such as high‐glucose conditions [58], and CRISPR‐based reporter systems for dynamic processes such as mitophagy further link tissue‐level functional outputs with subcellular mechanisms. Closed‐loop hiPSC‐derived cardiac tissue models and chamber‐specific engineered tissues provide additional examples of how electrical pacing, multicellular organization, and functional readouts can be integrated for drug assessment and disease modeling [59, 60].

Despite these advances, EHTs remain limited by cost, technical expertise requirements, batch‐to‐batch variability, and incomplete representation of endogenous vascular networks and immune components. Force readouts can also be affected by matrix stiffness, tissue geometry, cell composition, and maturation state, which complicates cross‐study comparison. These constraints have driven the field toward cardiac organoids and multicellular microphysiological platforms that more fully address tissue composition, spatial organization, and perfusion. Therefore, EHT‐based I/R studies should report not only survival or contractile recovery, but also tissue geometry, matrix composition or stiffness, cell composition, pacing conditions, and maturation status.

#### 3.1.3. Cardiac Organoids and Multicellular Platforms

Cardiac organoids and multicellular microphysiological platforms represent a major advance in I/R injury modeling because they can recapitulate selected multicellular, spatial, and diffusion‐limited features of human myocardial injury [16, 61]. In contrast to reductionist 2D systems, 3D cardiac organoids integrate cardiomyocytes with endothelial cells, fibroblasts, and epicardial‐like lineages, thereby enabling emergent tissue behaviors shaped by spatial organization, paracrine crosstalk, and diffusion‐limited metabolism, all of which are central to infarct evolution. Foundational infarct organoid models demonstrated that these microtissues can reproduce oxygen diffusion constraints and infarct‐like stress responses in a controllable human system [16].

A major technological advance has been the generation of vascularized and/or chambered architectures, which directly address oxygen transport and microenvironmental gradients, the biophysical basis of ischemic core formation and reperfusion heterogeneity. Vascularized cardiac organoids increase hypoxia tolerance and expand the dynamic range across which injury phenotypes can be modeled [62]. These architectural innovations are increasingly converging with perfusable heart‐on‐a‐chip systems, enabling controlled flow and spatiotemporally defined I/R protocols and creating new opportunities to interrogate drug delivery under physiologically relevant transport constraints [47].

Multicellular platforms have enabled modeling of the immune and remodeling dimensions of I/R injury [63]. Inflammatory ventricular organoids can capture coupled programs of parenchymal injury and immune activation [63], whereas immune–cardiac assembloids or immune‐enabled heart‐on‐a‐chip platforms incorporating macrophage‐like cells make it possible to test how immune polarization states influence cardiac structure and function [64, 65].

Organoid models can reproduce postinfarct‐like fibroblast activation, collagen deposition, and tissue stiffening, thereby offering a controllable human platform for investigating fibrotic progression after myocardial infarction [14]. Region‐resolved microfluidic chip models further reconstruct scar, border zone, and immune–fibrotic interactions within a single device [66]. However, the biological complexity of these systems creates trade‐offs: Organoids show greater self‐organized tissue behavior but often suffer from variability in size, composition, and maturation, whereas chip systems provide stronger environmental control but are lower throughput and more costly to standardize. Hybrid organoid–chip paradigms incorporating perfusion, vascular interfaces, mechanical loading, and integrated sensors may be especially useful for future translational studies, but their reproducibility and clinical predictive value still require systematic benchmarking [67, 68].

A related but distinct line of work involves engineered tissue sheet approaches for myocardial repair. Human adipose‐derived stem cell tissue sheets and composite hiPSC‐CM/hADSC tissue sheets have shown beneficial effects in myocardial infarction models [55, 69]. These studies are valuable for translational tissue engineering, but they should not be overinterpreted as direct evidence that hiPSC‐based in vitro I/R platforms can predict clinical cardioprotection. Instead, they provide complementary evidence that engineered human‐relevant cardiac tissues and stromal support systems can influence injury repair, paracrine signaling, angiogenesis, and functional recovery.

## 4. Emerging Technologies and Mechanistic Insights in hiPSC‐Based Myocardial I/R Models

### 4.1. Emerging Technologies That Redefine Mechanistic Resolution in hiPSC‐Based I/R Models

The integration of single‐cell multiomics, ST, and CRISPR–Cas9 functional genomics into hiPSC‐based I/R injury models is shifting the field from population‐averaged phenotyping toward a new paradigm: resolving injury heterogeneity at single‐cell resolution, mapping injury gradients within tissue space, and establishing causality through targeted genetic perturbation. The convergence of these three technologies creates a coherent experimental framework that moves investigation from descriptive observation to mechanistic validation.

#### 4.1.1. Single‐Cell RNA Sequencing (scRNA‐seq): Injury Heterogeneity and Vulnerable Subpopulations

Early hiPSC‐based I/R injury studies relying on bulk transcriptomics implicitly assumed relatively uniform cardiomyocyte injury. scRNA‐seq has challenged this assumption by revealing that I/R injury involves heterogeneous cardiomyocyte and nonmyocyte stress states.

First, scRNA‐seq has uncovered intrinsic heterogeneity in cardiomyocyte metabolic programs [70] and stress responses [71]. Subclusters differ in mitochondrial oxidative modules and redox‐buffering capacity, providing a mechanistic basis for stratified oxidative vulnerability during reperfusion [72]. Spatial multiomics studies of human infarct tissue further support this framework by demonstrating region‐specific cardiomyocyte stress states across infarct core and border zones, thereby reinforcing a model of state stratification rather than uniform injury [73].

Second, scRNA‐seq has clarified that inflammatory and cell death programs during reperfusion are often driven by discrete cell states [74]. Regulated death modalities such as ferroptosis may be embedded within defined multicellular immune–stromal niches, implying that injury frequently arises from coordinated intercellular responses rather than diffuse, cell‐autonomous failure [75]. In hiPSC‐based cardiac organoid H/R models, interferon/STAT‐associated programs have been identified as dominant injury axes rather than globally distributed signals [63]. Importantly, scRNA‐seq has also linked ferroptosis‐enriched vulnerable states to upstream regulators. For example, RBM15‐mediated m6A modification can stabilize ACSL4 and thereby promote ferroptosis in human cardiomyocytes, providing a concrete target for subsequent CRISPR‐based causal testing [76].

Collectively, scRNA‐seq reframes I/R injury as a condition of selective vulnerability: Cardiomyocyte subpopulations with specific metabolic configurations, limited redox reserve, and ferroptotic priming, positioned within particular inflammatory or vascular niches, may be more susceptible to failure. This state‐plus‐niche framework provides a conceptual roadmap for preclinical precision cardioprotection by shifting emphasis away from average protection of the entire myocardium toward targeted interrogation of vulnerable states and microenvironmental amplifiers.

#### 4.1.2. ST: Resolving Injury Gradients in 3D Cardiac Tissues

Although single‐cell approaches capture diversity in cellular states, they typically lose spatial context. ST addresses this limitation by anchoring transcriptional programs to defined coordinates within three‐dimensional tissues, thereby resolving injury gradients, border zone biology, and neighborhood relationships [77]. The value of ST is especially evident in hiPSC‐derived 3D cardiac organoids and engineered tissues [62], which inherently generate diffusion‐limited gradients of oxygen and nutrients. ST may enable such tissues to be deconstructed into hierarchical injury maps, including a peripheral hypoxic band, a central injury core, and a perinecrotic remodeling ring, each enriched for distinct molecular programs. This core–border–remote spatial organization closely mirrors patterns observed by spatial multiomics analyses of human myocardial infarction [72].

ST has also provided compelling evidence that many stress, inflammatory, and remodeling responses are spatially constrained. For instance, spatial multiomics analyses of AMI have revealed anatomically restricted routes of immune cell entry via the endocardium, underscoring that inflammatory interfaces are shaped by structural context [73]. Accordingly, ST enables hiPSC‐based I/R injury platforms to move beyond cell‐level phenotypes toward tissue‐scale mechanistic cartography. It localizes scRNA‐seq‐defined vulnerable states to specific ecological niches, thereby informing not only which pathway to target but also where within tissue space intervention may be most effective [78].

#### 4.1.3. CRISPR–Cas9 and Functional Genomics

Although scRNA‐seq and ST reveal complex heterogeneity and spatial patterning, many findings remain correlational. The integration of CRISPR–Cas9 and CRISPRi/a functional genomics into hiPSC platforms enables direct causal testing [79, 80]. Researchers can deploy genome‐wide CRISPRi/a screens or focused perturbations of candidate genes to validate regulatory nodes nominated by omics analyses [81]. For example, CRISPRi/a screens in hiPSC‐CMs have identified glycolytic activation as a druggable stress response pathway in doxorubicin‐induced cardiotoxicity, illustrating how functional genomics can nominate causal pathways that may be further tested in I/R‐specific platforms [80]. Such screens can be linked to survival, contraction, calcium handling, and metabolic readouts, offering a route from genetic target prioritization to pharmacological validation.

Perturb‐seq combines CRISPR perturbation with single‐cell transcriptomics to quantify how individual genetic perturbations rewire transcriptional networks and reshape cell‐state trajectories [82]. This approach can test whether disruption of an scRNA‐seq‐nominated candidate alters the abundance or fate of vulnerable subpopulations. CRISPR editing also supports investigation of individualized susceptibility through isogenic hiPSC lines differing only at defined variants [67]. Nevertheless, CRISPR‐based strategies require attention to editing efficiency, off‐target effects, cell‐state‐dependent fitness bias, and the limited scalability of complex 3D tissues. In multicellular hiPSC‐based I/R platforms, causal perturbation in nonmyocyte compartments such as fibroblasts or immune cells remains particularly important but technically demanding [83].

#### 4.1.4. Convergence of Artificial Intelligence (AI) and High‐Content Imaging (HCI): From Phenotypic Quantification to Predictive Modeling

The integration of AI with HCI is transforming hiPSC‐based I/R injury and H/R models from manually interpreted systems centered largely on static description into high‐throughput platforms capable of precise phenotypic deconvolution and dynamic prediction. HCI provides multiparametric, scalable cellular and subcellular readouts, whereas deep learning methods convert high‐dimensional images and videos into reproducible and comparable digital phenotypes. These phenotypes complement multiomics data and together generate convergent lines of mechanistic evidence [84].

##### 4.1.4.1. Quantifying Functional and Structural Phenotypes Beyond Manual Scoring

In large‐scale comparisons across batches and cell lines, manual annotation or simple threshold‐based algorithms often suffer from subjectivity and limited sensitivity to subtle phenotypes [85]. At the functional level, AI has advanced analysis beyond beat‐rate counting toward fine‐grained characterization of contraction dynamics and calcium signaling. Automated pipelines applied to bright‐field and fluorescence videos can simultaneously extract contraction waveforms, amplitude–velocity parameters, and calcium transient features, enabling sensitive detection of H/R‐induced excitation–contraction uncoupling and contractile phenotype drift [86]. These approaches improve sensitivity to mild dysfunction and support substantially higher throughput comparisons across compounds and experimental conditions [86].

At the subcellular level, a central pathological hallmark of reperfusion, namely, disrupted mitochondrial dynamics, can now be quantified with high precision. Deep learning–based segmentation of mitochondria in live‐cell imaging enables extraction of morphological descriptors, such as fragmentation and network connectivity, that correlate with functional state. This provides robust tools for quantifying reperfusion‐associated mitochondrial injury and its spatial heterogeneity.

##### 4.1.4.2. Discriminating Cell Death Modalities to Reduce Attribution Bias

H/R frequently activates multiple cell death programs, including ferroptosis and apoptosis, and reliance on single molecular markers may, therefore, bias mechanistic attribution. Morphology‐based image fingerprints derived from high‐content strategies such as Cell Painting, when coupled with deep learning classifiers, have been shown to distinguish distinct cell death modalities using phenotypic signatures alone [87].

##### 4.1.4.3. Building Predictive Models Toward Individualized and Generalizable Inference

For translational modeling, AI‐driven prediction aims to relate hiPSC phenotypic data to individual‐specific risk or therapeutic response. Machine learning frameworks trained on high‐throughput microelectrode array recordings from patient‐derived iPSC‐CMs have been used to define relationships among genotype, electrophysiological susceptibility, and drug risk, thereby supporting experimental stratification of cardiotoxicity liability and screening decisions [88]. However, these models require sufficiently large training sets, transparent feature extraction, external validation, and control of line‐to‐line and batch effects before they can support clinically meaningful inference. Computational approaches such as anomaly detection and representation learning are increasingly being applied to quantify these sources of variation and guide more robust experimental design.

Taken together, AI–HCI integration is reshaping hiPSC‐based I/R injury research by enabling automated quantification of functional and structural injury, multidimensional discrimination of cell death phenotypes, and computational integration of imaging‐derived phenotypes with single‐cell and spatial omics. The main limitation is not analytical capacity but evidence generalizability: Models trained on limited cell lines, culture protocols, or imaging systems may not transfer across laboratories unless benchmark datasets, standardized metadata, and independent validation cohorts are established.

Overall, the strongest experimental designs are likely to combine omics, perturbation, imaging, and functional readouts within the same platform. This integrated approach is especially important in I/R injury because viability alone may miss early and reversible changes in electrical stability, contractile force, calcium cycling, mitochondrial function, and metabolic state.

### 4.2. Mechanistic Insights From hiPSC‐Based Myocardial I/R Models

**Figure 3:**
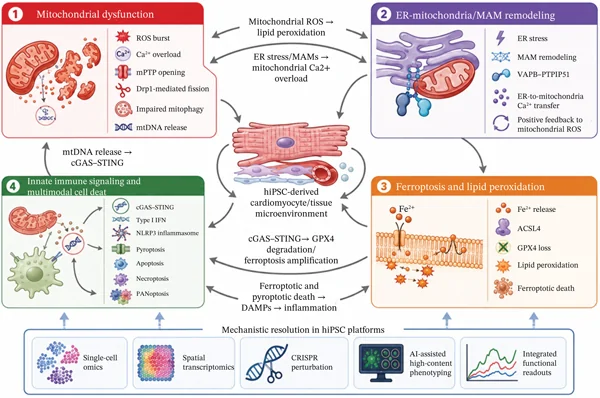
Integrated mechanisms and feedback loops in hiPSC‐based myocardial I/R models. The figure depicts mitochondrial ROS burst, Ca^2+^ overload, Drp1‐dependent fission, impaired mitophagy, ER stress, MAM remodeling, ferroptosis, lipid peroxidation, cGAS–STING activation, NLRP3 inflammasome signaling, pyroptosis, apoptosis, necroptosis, and PANoptosis as an interconnected network. It also illustrates how single‐cell/spatial omics, CRISPR perturbation, AI‐assisted phenotyping, and integrated functional readouts can interrogate these mechanisms in hiPSC‐based platforms. Some PANoptosis‐related mechanisms are inferred from myocardial I/R studies and remain to be systematically validated in hiPSC‐based platforms. DAMPs, damage‐associated molecular patterns; AI, artificial intelligence; ROS, reactive oxygen species; MAMs, mitochondria‐associated endoplasmic reticulum membranes.

Mechanistic interrogation using hiPSC platforms has advanced beyond the classical oxidative stress paradigm, revealing interconnected pathological axes that jointly shape reperfusion injury in a human cellular context (Figure 3). In this integrated framework, mitochondrial dysfunction, ferroptosis, ER–mitochondria crosstalk through mitochondria‐associated endoplasmic reticulum membranes (MAMs), cGAS–STING signaling, NLRP3 inflammasome activation, and multimodal cell death should not be viewed as isolated pathways. Instead, they form a reinforcing network in which mitochondrial Ca^2+^ overload and ROS production trigger mitochondrial DNA release, inflammatory signaling, lipid peroxidation, and further organelle damage. The sections below summarize representative evidence supporting these interacting injury modules.

#### 4.2.1. Mitochondrial Dynamics and Mitophagy

Disrupted mitochondrial dynamics and selective mitochondrial autophagy (mitophagy) constitute a tightly coupled axis linking energetic collapse, oxidative burst, and execution of cell death during reperfusion [89]. In this context, excessive Drp1 (DNM1L)‐dependent mitochondrial fission typically manifests as network fragmentation and is associated with ATP depletion and progressive accumulation of oxidative stress [90].

hiPSC platforms provide distinct opportunities for causal validation and target‐directed intervention in human cardiomyocytes and cardiac tissues. In human cardiac organoids engineered to model I/R, administration of a next‐generation Drp1 inhibitor during the reperfusion window reduced cell death, improved contractile function, and decreased mitochondrial superoxide signals. These phenotypic effects were accompanied by proteome‐level restoration of mitochondrial network integrity and metabolic pathways, providing direct pharmacodynamic validation at the level of human tissue [91]. Genetic evidence further supports this model: hiPSC‐CMs carrying pathogenic DNM1L variants exhibit abnormal mitochondrial morphology, impaired membrane potential and respiration, and secondary disturbances in Ca^2+^ dynamics and contractility, underscoring that the fission program itself is a critical determinant of human cardiomyocyte energy homeostasis [92].

Mitophagy pathways that counterbalance fission are widely regarded as a core endogenous protective mechanism for clearing damaged mitochondria and limiting catastrophic destabilization. A key methodological advance enabled by hiPSC systems is the real‐time quantification of mitophagy flux [93]. In hiPSC‐derived engineered myocardial tissues, pH‐sensitive mitochondrial reporters permit live tracking of acidified mitochondria during reperfusion, and perturbation of nodes such as ULK1, BNIP3, or PARKIN modulates mitochondrial delivery to lysosomes. This shifts analysis from descriptive observation toward pathway‐level dissection with greater mechanistic resolution [94].

Overall, by integrating live imaging, functional readouts, and genetic or pharmacological manipulation, hiPSC platforms are providing human‐relevant mechanistic validation and a translational rationale for mitochondria‐targeted strategies, including Drp1 inhibition and precise modulation of mitophagy [95].

#### 4.2.2. Ferroptosis and Lipid Peroxidation: A Systems Hub Linking Metabolism, Iron Homeostasis, and Innate Immunity

Accumulating evidence positions ferroptosis as a central mechanism of metabolic vulnerability in myocardial I/R injury. Its execution reflects increased susceptibility to membrane lipid peroxidation, disruption of intracellular iron homeostasis, and collapse of antioxidant defenses centered on the system Xc^−^–GSH–GPX4 axis [96, 97]. During reperfusion, abrupt oxygen restoration can trigger a burst of mitochondrial ROS and promote iron redox cycling, thereby driving ACSL4‐dependent enrichment of peroxidation‐prone polyunsaturated fatty acid–containing phospholipids and ultimately precipitating ferroptotic death [98].

Recent human‐relevant and translational studies, together with hiPSC‐CM H/R models, indicate that ferroptosis is closely coupled to innate immune signaling and organelle stress. A 2025 report showed that cGAS–STING signaling can promote cardiomyocyte ferroptosis by inducing autophagic degradation of GPX4, thereby disabling lipid peroxide detoxification and amplifying reperfusion injury [99]. Mechanistically, STING enhanced autophagosome–lysosome fusion and accelerated GPX4 proteolysis, forming a positive‐feedback loop that links inflammatory signaling to ferroptotic execution. Complementary adipose tissue–derived paracrine strategies provide tissue‐level evidence that cardioprotection can be achieved through mechanisms extending beyond cardiomyocyte‐intrinsic pathways, some of which involve ferroptosis inhibition. Brown adipocyte sheets were reported to alleviate myocardial I/R injury through NRG4–ErbB4‐dependent inhibition of ferroptosis [100]. Separately, brown adipose tissue–derived small extracellular vesicles were shown to mediate exercise‐associated cardioprotection after myocardial I/R injury, at least in part through vesicle‐associated miRNAs and suppression of proapoptotic signaling [101]. These findings support adipose tissue–derived paracrine cardioprotection, but their relevance to direct ferroptosis regulation in human cardiomyocytes should be validated in standardized hiPSC‐CM H/R, EHT, cardiac organoid, and heart‐on‐a‐chip I/R platforms. At the regulatory level, ACSL4 is widely regarded as a pivotal determinant of ferroptosis sensitivity [98]. Recent work has proposed that the Parkin–ACSL4 axis may link mitochondrial quality control to lipid peroxidation susceptibility, suggesting a potential connection between mitophagy‐related regulation and ferroptosis control [102]. Perturbations in iron handling are also central drivers of this process [103]. Mitochondria play a dual role in ferroptosis control: Mitochondrial ROS production promotes injury, whereas the mitochondrial DHODH–CoQ10 and cytosolic FSP1–CoQ10 pathways provide GPX4‐independent layers of antioxidant defense [104, 105].

Taken together, ferroptosis emerges as a systems‐level mechanistic nexus connecting mitochondrial dysfunction, ER stress, iron dysregulation, lipid remodeling, and innate immune activation. In hiPSC‐CM H/R models, genetic or pharmacological manipulation of GPX4, ACSL4, or iron‐handling genes enables dissection of ferroptosis vulnerability in a human cellular context. At the same time, many ferroptosis‐targeted protective effects still rely on animal transplantation or non‐hiPSC systems, so their relevance to patient‐specific cardioprotection requires further validation in standardized hiPSC‐CM, EHT, organoid, and chip‐based I/R models.

#### 4.2.3. ER Stress–Mitochondria Crosstalk

In hiPSC‐based I/R injury models, MAMs are increasingly recognized as integrative hubs that causally couple ER proteostasis collapse, particularly activation of the unfolded protein response (UPR), to mitochondrial Ca^2+^ overload, redox imbalance, and apoptosis. This has enabled mechanistic dissection of H/R injury cascades in human cardiomyocytes [106, 107]. H/R imposes a combined burden of protein folding stress and energetic crisis that activates the three canonical UPR branches. Early signaling may be adaptive; however, persistent stress drives pro‐death transcriptional programs and lowers the mitochondrial threshold for Ca^2+^‐ and ROS‐triggered failure. Crucially, ER stress is not merely coincident with mitochondrial dysfunction. By remodeling the structure and flux properties of MAMs, it amplifies ER‐to‐mitochondria Ca^2+^ transfer, driving mitochondrial Ca^2+^ overload, mtROS burst, increased mPTP susceptibility, and energetic collapse. These events can in turn intensify ER oxidative folding stress, thereby establishing a positive‐feedback loop across the ER–MAM–mtROS axis [108].

Recent myocardial I/R studies provide intervention‐oriented evidence that MAM remodeling can be pharmacologically targeted. These findings provide mechanistic hypotheses that can be further tested in standardized hiPSC‐CM, EHT, organoid, and chip‐based H/R platforms. The PERK agonist ZY341 was reported to confer protection in cardiomyocyte H/R models by suppressing pathological MAM expansion, modulating the VAPB/PTPIP51 tethering complex, and normalizing mitochondrial Ca^2+^ flux and phosphatidic acid homeostasis. These findings support a pharmacological axis linking PERK activation to tether remodeling, Ca^2+^ and lipid flux control, and enhanced mitochondrial stress resistance [109]. Complementary work identified RHOA as a determinant of the abundance of mitochondria–endoplasmic reticulum contact sites through regulation of VAPB/PTPIP51 assembly and implicated upstream CUL3‐mediated control, thereby providing more proximal leverage points for structure‐oriented MAM‐targeting strategies [110].

Notably, MAM‐associated Ca^2+^ microdomains may also participate in systems‐level amplification of reperfusion injury across metabolism, immunity, and the microvasculature. Lactate‐driven upregulation of MICU3, coupled with VDAC1‐associated MAM formation, can promote excessive mitochondrial Ca^2+^ uptake, mitochondrial dysfunction, and impaired autophagy while facilitating neutrophil extracellular trap–associated microvascular injury. This links metabolic cues to Ca^2+^–ROS pathology at a broader systems level [111]. In 3D hiPSC cardiac microtissues and organoids, diffusion‐limited oxygen and metabolic gradients generate marked spatial heterogeneity, allowing ER stress–mitochondria crosstalk to be resolved along core–edge and necrotic–border axes rather than being obscured by 2D or bulk‐averaged readouts [112, 113].

Overall, emerging evidence is moving MAMs from phenomenological contact sites toward pharmacologically and genetically tractable target domains. For hiPSC‐based platforms, the key opportunity is to connect MAM structure, Ca^2+^ flux, mitochondrial energetics, and tissue‐level functional recovery rather than treating ER stress markers as isolated endpoints [114, 115].

#### 4.2.4. The NLRP3 Inflammasome and Innate Immune Signaling

In immune‐competent human cardiac microtissues and organoids generated on hiPSC platforms, innate immune signaling is no longer viewed solely as a secondary consequence of infarction but rather as a core injurious module that can arise early during reperfusion and reciprocally amplify parenchymal damage. H/R‐driven mitochondrial collapse and the release of damage‐associated molecular patterns provide NF‐*κ*B‐dependent priming signals, whereas ionic disequilibrium and mitochondrial stress trigger NLRP3 assembly and Caspase‐1 activation, promoting maturation and release of IL‐1*β* and IL‐18 and inducing gasdermin D–mediated pyroptosis. In this way, local inflammation shifts from a signaling process to a cell death–driven amplifier [116, 117].

A key advance is the demonstration in macrophage‐integrated human cardiac microtissues and organoids that, within multicellular contexts, macrophages can markedly intensify inflammasome‐associated phenotypes through paracrine IL‐1 family signaling, dysregulated efferocytosis, and remodeling of electrical and matrix coupling, thereby establishing persistent immune–myocardial feedback circuits. These findings suggest that cardiomyocyte‐only hiPSC‐CM monocultures systematically underestimate the injurious contribution of inflammasome signaling [118].

In parallel, the cooperation between cytosolic nucleic acid sensing and the NLRP3 inflammasome is increasingly being consolidated into a unified framework. During reperfusion, mitochondrial damage can enable cytosolic mtDNA‐driven STING activation. Downstream IRF3 programs induce Type I interferons and interferon‐stimulated genes that not only increase border zone fragility but can also create positive feedback with inflammasomes and multiple cell death pathways, escalating injury from cytokine‐driven inflammation to a secondary interferon–cell death amplification module [119, 120]. Consistent with this view, reperfusion‐context studies suggest that STING can couple to metabolic–ferroptosis axes through selective autophagy and proteostasis mechanisms and can also interface with inflammasome–pyroptosis networks, helping to explain why single‐node blockade often fails to fully suppress the composite injury spectrum of human H/R models [99].

More broadly, recent work elevates inflammatory–cell death crosstalk into the unified paradigm of PANoptosis and PANoptosome signaling [121, 122]. In myocardial I/R, innate immune sensors such as ZBP1 can promote noncanonical PANoptosome assembly under H/R, coordinating pyroptosis, apoptosis, and necroptosis to jointly determine infarct expansion and the magnitude of reperfusion injury [116]. In the context of translational failure, immune‐integrated hiPSC organoid and microphysiological systems can simultaneously track parenchymal injury, inflammasome–pyroptosis signaling, cGAS–STING–interferon amplification, and multimodal cell death coupling. These features may help define efficacy boundaries and combination windows for anti‐inflammatory or immunomodulatory cardioprotective strategies, but they require careful validation against human tissue and clinical data.

#### 4.2.5. Integrated Pathway Model: Interconnected Injury Loops

Reoxygenation increases mitochondrial ROS production, mitochondrial Ca^2+^ overload, mPTP susceptibility, and mitochondrial fission, thereby promoting mitochondrial dysfunction and mtDNA release [89–95]. ER stress and MAM remodeling further amplify ER‐to‐mitochondria Ca^2+^ transfer and reinforce mitochondrial ROS production [106–115]. In parallel, iron dysregulation, ACSL4‐dependent lipid remodeling, GPX4 loss, and ferritinophagy increase lipid peroxidation and ferroptosis susceptibility [96–105]. Mitochondrial damage can activate cGAS–STING signaling, interferon responses, and NLRP3 inflammasome pathways, whereas ferroptotic, pyroptotic, apoptotic, and necroptotic programs can release damage signals that further amplify inflammation [99, 116–122]. Thus, mitochondrial dysfunction, MAM remodeling, ferroptosis, and innate immune signaling form mutually reinforcing injury loops rather than independent pathways.

## 5. Translational Applications, Current Limitations, and Future Directions (Figure 4)

**Figure 4:**
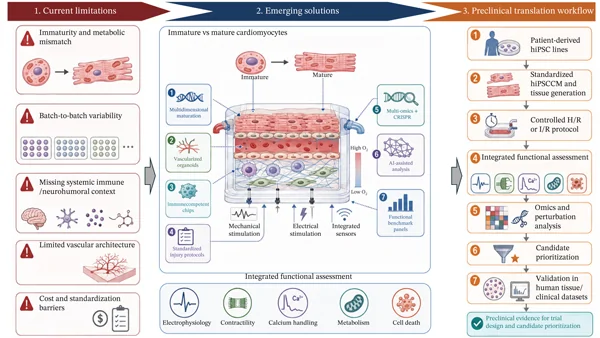
Current limitations, emerging solutions, and future translational workflow for hiPSC‐based myocardial I/R platforms. The figure presents a future‐oriented workflow that moves beyond maturation alone by integrating multicellular cardiac tissues, vascularization, immune competence, disease modeling, multiomics, CRISPR‐based functional genomics, AI‐assisted analysis, integrated electrophysiology–contractility–calcium–metabolism assessment, and patient‐specific preclinical modeling. Immature and mature cardiomyocytes are represented as cells rather than small and large hearts.

### 5.1. Translational Applications: Drug Discovery, Safety Assessment, and Preclinical Precision Modeling

hiPSC‐based cardiac models are evolving from experimental research tools into preclinical platforms that can help bridge the gap between mechanistic discovery and translational testing. Their applications include mechanism‐guided drug screening, safety and liability assessment, and patient‐specific experimental modeling. However, these applications remain primarily preclinical. Claims regarding clinical risk stratification or individualized cardioprotection should, therefore, be framed as future possibilities requiring large‐scale validation rather than established clinical utility.

#### 5.1.1. Cardioprotective Drug Screening

hiPSC‐based cardiac systems are becoming an important screening platform in I/R injury research by enabling scalable phenotypic discovery in a human cellular context. In 2D hiPSC‐CM H/R assays, investigators can quantify multiple functional dimensions, including contractility, calcium transient kinetics, metabolic state, ROS burden, and cell death fraction, to assemble an integrated anti‐ischemic phenotype that supports systematic comparison and prioritization of candidate compounds [12, 123].

##### 5.1.1.1. Screening Mitochondria‐Targeted Cardioprotective Strategies

hiPSC‐CM models can be configured to track mitochondrial membrane potential, calcium overload, oxidative stress, and contractile function, thereby providing a mechanistically anchored framework for evaluating mitochondrial stabilizers and candidate mPTP‐modulating strategies. Similarly, ferroptosis‐focused discovery can incorporate lipid peroxidation, GPX4–system Xc^−^ activity, and iron homeostasis readouts to distinguish general antioxidant effects from suppression of lipid radical chain propagation.

##### 5.1.1.2. Multicellular and Perfused Platforms Increase Physiological Relevance

Because reperfusion injury is shaped by complex intercellular interactions, more advanced systems are increasingly being used for screening. Multicellular hiPSC‐derived heart organoids containing cardiomyocytes, fibroblasts, and endothelial cells can reproduce key features of AMI and I/R‐associated injury, including cell death, altered calcium handling, beating abnormalities, and fibrotic remodeling [14, 16]. Multicellular cardiac organoids incorporating macrophage‐like cells provide a more physiologically relevant setting for anti‐inflammatory drug discovery. For example, in immune‐containing ventricular organoid I/R models, Type I interferon signaling has been implicated as an injury‐amplifying axis, and IFNAR blockade markedly reduced inflammatory outputs and oxidative stress, illustrating the potential of human organoid platforms for mechanism‐driven therapeutic discovery [63]. In parallel, vascularized and perfusable heart‐on‐a‐chip and related microphysiological I/R systems show how controlled perfusion, oxygen gradient manipulation, and multicellular microenvironments can be used to evaluate cardioprotective interventions under controlled injury conditions [47, 124].

Chip‐based systems cultured under fluid shear and incorporating multiple cell types indicate that key nonmyocyte compartments substantially reshape the trajectory of postreperfusion cell death and tissue‐level functional change, underscoring the importance of multicellular microenvironments in screening and prioritization [63, 124].

##### 5.1.1.3. AI‐Enabled High‐Content Screening for Precision Cardioprotection

As automated production of hiPSC‐derived 3D cardiac microtissues converges with high‐throughput functional testing, both the scale and dimensionality of cardioprotective screening are expanding. Multimodal outputs, such as calcium signaling combined with electrophysiological parameters, enhance prediction of proarrhythmic liability and positive or negative inotropic effects [125]. AI and machine learning approaches are increasingly applied to microelectrode array recordings, calcium transient traces, contraction videos, and imaging data to model dose–response relationships and prioritize compound libraries [126]. In patient‐derived hiPSC‐CMs, these methods may support experimental drug response stratification, but clinical prediction requires larger biobanks, standardized metadata, and prospective validation.

#### 5.1.2. Cardiotoxicity Assessment

The interface between cardiotoxicity assessment and ischemic heart disease research is becoming increasingly important, because the toxic mechanisms of many anticancer therapies (e.g., anthracyclines and tyrosine kinase inhibitors)—including oxidative stress, mitochondrial dysfunction, and inflammation—overlap substantially with core I/R injury pathology [127, 128].

A distinct advantage of hiPSC‐based I/R injury platforms is their ability to capture early reperfusion‐specific injury events, including an immediate ROS burst after reoxygenation, mPTP opening, intracellular Ca^2+^ overload, and energetic collapse, which often precede overt cardiomyocyte death [129–131]. These platforms may complement classical cardiotoxicity assays by testing whether drug exposure increases susceptibility to reperfusion‐relevant stress phenotypes, including ROS burst, calcium overload, mPTP opening, energetic failure, and impaired contractile recovery.

#### 5.1.3. Patient‐Derived Precision Modeling: Preclinical Opportunities and Evidence Boundaries

Patient‐specific hiPSC platforms provide an experimental route for studying how human genetic background influences susceptibility to I/R injury and therapeutic responsiveness [132, 133]. Because hiPSCs preserve donor‐specific genetic variation, patient‐derived hiPSC‐CMs can be used under controlled conditions to map genotype–phenotype relationships. This is a clear advantage over conventional animal models or immortalized cell lines with limited genetic diversity. Nevertheless, the current evidence mainly supports preclinical modeling and hypothesis generation, not routine clinical decision‐making.

One important application is functional dissection of genetic variants that modulate cardiomyocyte stress vulnerability. Patient‐derived hiPSC‐CM models carrying genome‐wide association study–nominated risk variants (e.g., variants associated with myocardial infarction risk or mitochondrial dysfunction) can be used to define how genetic background alters key injury events during H/R [134]. Coupling these models to CRISPR editing enables causal testing of candidate susceptibility genes, thereby revealing genetic networks that govern I/R responses [135, 136].

A second application is the study of interindividual differences in drug response. Studies using patient‐derived or genetically diverse hiPSC‐CM panels suggest that donor background can influence cellular phenotypes and drug responses. When combined with high‐content phenotyping and machine learning analytics, such datasets may help identify response patterns and candidate responder subgroups [133, 135]. However, I/R‐specific biobank validation remains limited, and prospective stratification of responders and nonresponders before clinical testing remains an aspirational goal requiring standardized assays, sufficiently powered biobanks, clinical metadata, and external validation.

Overall, patient‐derived hiPSC platforms provide a useful preclinical bridge for evaluating cardioprotective strategies under human genetic contexts. Their most immediate value lies in mechanistic stratification, target prioritization, and improved trial design rather than direct individualized therapy selection. This evidence boundary is important because overstatement of precision cardioprotection could obscure the substantial validation steps still required before clinical implementation. Therefore, patient‐derived hiPSC platforms should currently be viewed as tools for preclinical responder hypothesis generation rather than as validated assays for selecting cardioprotective therapy in individual patients.

#### 5.1.4. Integrated Functional Assessment in Advanced Engineered Cardiac Platforms

A major requirement for translationally useful I/R platforms is simultaneous or coordinated assessment of electrophysiology, contractility, calcium handling, mitochondrial function, and metabolic activity. Reperfusion injury does not manifest as a single endpoint: Electrical instability, impaired excitation–contraction coupling, delayed calcium reuptake, reduced contractile recovery, ROS burst, ATP depletion, and regulated cell death can occur at different times and with different drug sensitivities. Therefore, platform value increases when multiple functional domains are measured in parallel rather than inferred from viability alone.

Engineered cardiac tissues and heart‐on‐a‐chip systems increasingly support such integrated assessment. Chamber‐specific engineered cardiac tissues enable controlled pacing and drug testing while capturing atrial‐ and ventricular‐like functional differences [60]. At the hiPSC‐CM assay level, simultaneous optical measurement of action potential, cytosolic calcium, and contraction has shown that integrated excitation–contraction coupling readouts can identify mechanisms underlying contractile drug responses more sensitively than isolated endpoints [137]. Hydrogel‐free heart tissue platforms with dual excitation–contraction recording provide a route for linking electrophysiological behavior to mechanical output [138]. Biosensing platforms that simultaneously record contraction force and field potentials can further reveal whether a compound primarily affects electrical conduction, mechanical contraction, or their coupling [139]. For I/R studies, these approaches are especially relevant because a treatment that improves survival but worsens arrhythmogenicity or contractile recovery may not be translationally useful.

Future benchmark studies should, therefore, report a minimum functional panel that includes electrophysiological indices, contractile amplitude or force, calcium transient amplitude and decay, mitochondrial membrane potential or respiration, ROS production, and cell death markers. Standardized multiparametric readouts would improve cross‐platform comparison and reduce the risk of overinterpreting single‐endpoint cardioprotection. Such multiparametric assessment is particularly important because apparent cardioprotection based on survival alone may conceal persistent electrical instability, impaired calcium reuptake, or poor contractile recovery.

### 5.2. Current Limitations of hiPSC‐Based Myocardial I/R Modeling

Despite substantial progress, hiPSC‐based I/R models retain fundamental limitations that directly constrain mechanistic depth and translational credibility.

#### 5.2.1. Immaturity and Metabolic Mismatch

The most frequently cited limitation of hiPSC‐CMs in in vitro I/R injury modeling remains their fetal‐like phenotype and metabolic profile. These cells typically rely predominantly on glycolysis, exhibit lower mitochondrial density and respiratory chain capacity, and display a depolarized resting membrane potential together with underdeveloped T‐tubules and calcium‐handling microarchitecture. Collectively, these features create a pronounced metabolic mismatch: Under I/R stress, substrate utilization and redox responses in the model do not faithfully recapitulate the state of adult myocardium dominated by fatty acid oxidation and oxidative phosphorylation [140].

Insufficient metabolic maturation not only complicates quantification of injury magnitude but may also alter the kinetics and thresholds of key injury pathways. For example, the timing and peak amplitude of the reperfusion ROS burst, the pool of oxidizable membrane lipids, and the coupling structure of iron homeostasis networks may shift systematically with developmental state [141]. This issue is particularly relevant to ferroptosis‐related mechanisms, which depend on lipid peroxidation substrate availability and multilayer antioxidant gatekeeper systems. Because these gatekeepers are tightly coupled to metabolic state, membrane lipid composition, and mitochondrial integrity, ferroptosis sensitivity and regulatory thresholds in metabolically immature hiPSC‐CMs may differ substantially from those in adult myocardium [142, 143].

#### 5.2.2. Batch‐to‐Batch Variability

Batch‐to‐batch variability is widely recognized as a major technical barrier to reproducibility and cross‐laboratory consistency in hiPSC‐based I/R injury research. This variability includes differences among hiPSC lines as well as variation across differentiation batches within the same line. Such heterogeneity can substantially influence maturation state, metabolism, electrophysiology, and stress response programs, thereby systematically affecting I/R phenotypes and cardioprotective screening outcomes [144].

Donor genetic background and epigenetic memory both contribute to this variability, as do de novo genetic alterations arising during reprogramming or prolonged culture, and differences in cardiomyocyte differentiation efficiency and maturation [145]. Importantly, batch effects can distort mechanistic interpretation: For instance, differences in mitochondrial network maturation can shift ROS production kinetics and mPTP sensitivity, thereby influencing the apparent efficacy of mitochondria‐targeted interventions. Without systematic quality control, batch variability can, therefore, generate both false‐positive and false‐negative conclusions in cardioprotection studies.

#### 5.2.3. Absence of Systemic Immune and Neurohumoral Context

hiPSC‐based I/R injury platforms lack the complex systemic immune responses and neurohumoral regulation present in vivo. Clinically, I/R injury after AMI unfolds within a systemic pathophysiological context involving circulating inflammation and multiorgan regulation. Myocardial injury rapidly releases danger‐associated molecular patterns and activates innate immune pathways that drive recruitment of circulating immune cells and downstream inflammatory cascades [146]. However, most in vitro hiPSC‐based I/R injury models lack sustained influx of circulating immune cells and system‐level communication among immune organs, thereby limiting faithful reconstruction of these processes.

Neurohumoral systems are likewise central regulators of I/R injury. After AMI, the sympathetic nervous system and the renin–angiotensin–aldosterone system are rapidly activated and influence injury progression by modulating vascular tone, metabolic substrate utilization, inflammatory signaling, and electrophysiological stability [147]. Yet hiPSC cardiac models typically operate in relatively closed culture environments without circulating hormones, neural inputs, or multiorgan feedback loops. Future integration of cardiac tissues with immune modules, vascularized microphysiological systems, and multiorgan organ‐on‐chip platforms may improve reconstruction of the systemic AMI‐associated I/R injury pathology and enhance translational value.

#### 5.2.4. Insufficient Tissue Architecture and Vascular Complexity

Although EHTs, cardiac organoids, and heart‐on‐a‐chip technologies have markedly increased tissue complexity in hiPSC models, most systems still fall short of reproducing the spatial architecture and vascular organization of the adult human heart [148]. Native myocardium comprises cardiomyocytes, endothelial cells, fibroblasts, immune cells, and other lineages embedded within a highly organized extracellular matrix and hierarchical vascular tree, forming an integrated functional structure through mechanical coupling, paracrine signaling, and metabolic gradients. By contrast, most current hiPSC‐derived models remain dependent on diffusion for oxygen and nutrient exchange, limiting tissue size and architectural complexity.

For I/R injury studies, the absence of a perfusable vascular network is a particularly consequential limitation [148]. In vivo, coronary circulation not only maintains oxygen and substrate delivery but also governs restoration of local oxygen gradients, endothelial barrier function, and microcirculatory perfusion during reperfusion—processes central to injury regulation [148]. Yet most in vitro myocardial models cannot reproduce physiological hemodynamics or key pathological features such as microvascular dysfunction [148].

Recent efforts have sought to address this gap through microfluidic perfusion, vascularized organoids, and heart‐on‐a‐chip systems. For example, incorporation of endothelial cells into microfluidic cardiac chips can induce endothelial polarization and tight junction formation under fluid shear, approximating aspects of vascular barrier function [18]. In addition, vascularized cardiac organoids and vascularized cardiac spheroid‐on‐a‐chip systems incorporating endothelial components can partially reconstitute local microvascular‐like networks and improve tissue organization or maturation [62, 149, 150]. Nonetheless, these systems largely remain at the level of local microvascular units and do not yet reproduce the heart′s hierarchical vascular tree or multiscale hemodynamic regulation. Thus, constructing vascularized myocardial models with stable perfusion capacity and physiologically realistic flow dynamics remains a major technical frontier in hiPSC‐based I/R injury research.

#### 5.2.5. Reproducibility, Scalability, Cost, and Standardization

Beyond biological limitations, practical barriers substantially constrain the translational use of hiPSC‐based I/R platforms. Differentiation efficiency, cardiomyocyte purity, maturation status, oxygen‐switching protocols, substrate composition, pacing conditions, matrix stiffness, tissue geometry, and endpoint timing vary substantially across laboratories. These differences can alter injury severity and drug response magnitude, making cross‐study comparison difficult.

Scalability and cost also vary by platform. 2D H/R assays are relatively inexpensive and compatible with high‐throughput screening, but they are structurally simplified. EHTs and organoids provide richer tissue‐level information but require more cells, longer culture periods, more specialized equipment, and more complex quality control. Heart‐on‐a‐chip systems offer precise control of perfusion and oxygen gradients but remain technically demanding and comparatively low‐throughput. Broad translation will, therefore, require consensus reporting standards, reference hiPSC lines, shared injury protocol benchmarks, standardized maturation metrics, and external validation across laboratories. At a minimum, future studies should report hiPSC line information, passage number, differentiation efficiency, cardiomyocyte purity, maturation protocol, substrate composition, oxygen level and duration, reoxygenation timing, pacing or loading conditions, cell composition, and predefined functional endpoints.

### 5.3. Emerging Solutions and Future Directions

Despite rapid progress in hiPSC‐based cardiac disease modeling, bridging the translational gap in myocardial I/R injury will require advances at multiple technical and conceptual levels. Next‐generation platforms must improve maturation, vascularization, immune competence, functional integration, and cross‐laboratory reproducibility. Progress in stem cell engineering, biofabrication, multiomics analytics, and computational biology is expected to improve mechanistic discovery and therapeutic screening, but clinical translation will depend on rigorous validation rather than platform complexity alone.

#### 5.3.1. Advancing Cardiomyocyte and Tissue Maturation and Complexity

A central limitation of current hiPSC‐derived cardiomyocyte systems remains their fetal‐like immature phenotype [151]. Because susceptibility to I/R injury is highly dependent on mitochondrial oxidative capacity and calcium‐handling efficiency, improving cardiomyocyte maturation is essential for enhancing physiological relevance. Evidence indicates that integrating multidimensional maturation strategies—including fatty acid–based metabolic reprogramming, long‐term electrical stimulation, mechanical loading, and perfused microphysiological culture—can substantially improve the structural and metabolic maturation of engineered myocardial tissues. In addition, microfluidic heart‐on‐a‐chip platforms can provide sustained delivery of nutrients and oxygen together with mechanical cues, thereby recreating key physiological signals that shape the myocardium in vivo [17, 38, 47, 152]. Looking ahead, advanced fabrication and biomaterial‐guided tissue construction may enable the generation of engineered myocardium with anisotropic architecture and tailored mechanical properties, bringing both structure and function closer to those of adult human heart tissue.

#### 5.3.2. Next‐Generation Models Incorporating Vascularization and Immune Competence

I/R injury is a systems‐level pathological process driven by complex interactions among cardiomyocytes, endothelial cells, fibroblasts, and immune cells [130, 153]. Yet many hiPSC‐derived cardiac models still lack functional vascular structures and immune cell components, limiting their capacity to reconstruct reperfusion‐associated inflammation and microvascular dysfunction [154, 155]. Accordingly, next‐generation cardiac models are increasingly moving toward vascularized and immune‐integrated organoid paradigms. Vascularized cardiac organoids containing lumenized endothelial structures and microvascular‐like networks have been shown to improve oxygen diffusion and enhance metabolic stability [62, 150]. In parallel, vascularized and perfusable heart‐on‐a‐chip systems allow controlled perfusion and analysis of I/R‐related transport constraints [47].

In parallel, incorporating immune cells—such as macrophages—into cardiac organoid systems is beginning to recapitulate inflammatory signaling networks that accompany I/R. Human heart‐on‐a‐chip studies have also shown that endothelial extracellular vesicle–mediated paracrine signaling can modulate I/R‐related injury in a perfused human microphysiological context [156]. Future convergence of cardiac organoids with organ‐on‐chip technologies is expected to enable precisely controlled perfusion, defined oxygen gradient landscapes, and programmable immune cell recruitment.

#### 5.3.3. Integrating Spatial Multiomics With Functional Genomics

Single‐cell and spatial multiomics studies have revealed pronounced heterogeneity in myocardial injury responses across cell states and tissue regions [72]. ST analyses of human myocardial infarction have demonstrated distinct transcriptional programs and cellular niches across the infarct core, border zone, and remote myocardium [72, 73], underscoring the importance of tissue architecture in shaping injury responses. Future work will likely integrate spatial multiomics more closely with CRISPR‐based functional genomics. For example, Perturb‐seq and pooled CRISPR screens can systematically validate candidate regulators identified by single‐cell or spatial profiling [157]. By combining spatially localized cell states with genetic perturbation experiments, investigators should be able to delineate causal regulatory networks governing mitochondrial dysfunction, ferroptosis susceptibility, and amplification of inflammatory signaling, thereby identifying new cardioprotective targets [72, 158–160].

#### 5.3.4. Toward Patient‐Specific Precision Cardioprotection

Patient‐specific disease modeling and precision medicine research remain compelling long‐term applications of hiPSC platforms [135, 161, 162]. Because hiPSC‐CMs preserve donor genetic background, they enable direct experimental assessment of how genetic variation may shape susceptibility to I/R injury and cardioprotective response [4]. As large‐scale hiPSC line libraries and biobanks expand, it should become increasingly feasible to quantify interindividual differences in mitochondrial metabolism, inflammatory signaling, and regulated cell death pathways. Integration with clinical genomic datasets and AI‐driven analytics may eventually support precision cardioprotection, but this goal remains dependent on standardized assays, large cohorts, and prospective validation.

## 6. Conclusion

hiPSC‐based cardiac platforms have expanded myocardial I/R injury research beyond animal models and immortalized cell lines toward systems with stronger human relevance and greater mechanistic tractability. Across a continuum from 2D monolayers to EHTs, organoids, and perfused heart‐on‐a‐chip systems, these models recapitulate selected pathological features of reperfusion injury while enabling controlled perturbation of mitochondrial dysfunction, ferroptosis, ER–mitochondria crosstalk, cGAS–STING signaling, inflammasome activation, and multimodal cell death.

Their principal current strength is not full replication of the adult human heart but controlled human‐relevant modeling of defined injury mechanisms and functional phenotypes. Mechanism‐guided screening, high‐content phenotyping, integrated electrophysiology–contractility–calcium–metabolism readouts, and patient‐derived hiPSC panels can improve target prioritization and preclinical drug assessment. However, incomplete maturation, batch variability, limited vascular and systemic immune complexity, high cost, and incomplete standardization continue to limit clinical predictiveness.

Future progress will depend on combining better tissue engineering with reproducible injury protocols, standardized quality control metrics, transparent computational models, and validation against human tissue and clinical datasets. If these constraints are progressively addressed, hiPSC‐based systems may become increasingly useful for preclinical cardioprotection research, patient subgroup hypothesis generation, and rational design of translational studies, whereas claims of established clinical precision cardioprotection should remain appropriately cautious.

## Author Contributions

All authors contributed to the conception, literature review, and writing of the manuscript.

## Funding

No funding was received for this manuscript.

## Disclosure

All authors have read and approved the final version.

## Ethics Statement

The authors have nothing to report.

## Conflicts of Interest

The authors declare no conflicts of interest.

## Data Availability

The authors have nothing to report.
